# In vivo characterisation of field pea stem wall thickness using optical coherence tomography

**DOI:** 10.1186/s13007-023-01075-1

**Published:** 2023-10-11

**Authors:** Qi Fang, Felipe A. Castro-Urrea, Felix Haederle, Rowan W. Sanderson, Dilusha Silva, Wallace A. Cowling, Brendan F. Kennedy

**Affiliations:** 1 Department of Electrical, Electronic & Computer Engineering, School of Engineering, The University of Western Australia, Crawley, WA 6009 Australia; 2 Centre for Medical Research, BRITElab, Harry Perkins Institute of Medical Research, QEII Medical Centre, The University of Western Australia, Crawley, WA 6009 Australia; 3 The UWA Institute of Agriculture, The University of Western Australia, Crawley, WA 6009 Australia; 4 School of Agriculture and Environment, The University of Western Australia, Crawley, WA 6009 Australia; 5 Australian Research Council Centre for Personalised Therapeutics Technologies, Melbourne, VIC Australia; 6 Institute of Physics, Nicolaus Copernicus University, Torun, Poland

**Keywords:** Field pea, Stem structure, Stem wall thickness, In vivo measurement, Optical coherence tomography

## Abstract

**Background:**

Modern field pea breeding faces a significant challenge in selecting lines with strong stems that resist lodging. Traditional methods of assessing stem strength involve destructive mechanical tests on mature stems after natural senescence, such as measuring stem flexion, stem buckling or the thickness of dry stems when compressed, but these measurements may not correspond to the strength of stems in the living plant. Optical coherence tomography (OCT) can be used as a noncontact and nondestructive method to measure stem wall thickness in living plants by acquiring two- or three-dimensional images of living plant tissue.

**Results:**

In this proof-of-principle study, we demonstrated in vivo characterisation of stem wall thickness using OCT, with the measurement corrected for the refractive index of the stem tissue. This in vivo characterisation was achieved through real-time imaging of stems, with an acquisition rate of 13 milliseconds per two-dimensional, cross-sectional OCT image. We also acquired OCT images of excised stems and compared the accuracy of in vivo OCT measurements of stem wall thickness with ex vivo results for 10 plants each of two field pea cultivars, Dunwa and Kaspa. In vivo OCT measurements of stem wall thickness have an average percent error of − 3.1% when compared with ex vivo measurements. Additionally, we performed in vivo measurements of both stem wall thickness and stem width at various internode positions on the two cultivars. The results revealed that Dunwa had a uniform stem wall thickness across different internode positions, while Kaspa had a significantly negative slope of − 0.0198 mm/node. Both cultivars exhibited an increase in stem width along the internode positions; however, Dunwa had a rate of increase of 0.1844 mm/node, which is three times higher than that of Kaspa.

**Conclusions:**

Our study has demonstrated the efficacy of OCT for accurate measurement of the stem wall thickness of live field pea. Moreover, OCT shows that the trends of stem wall thickness and stem width along the internode positions are different for the two cultivars, Dunwa and Kaspa, potentially hinting at differences in their stem strength. This rapid, in vivo imaging method provides a useful tool for characterising physical traits critical in breeding cultivars that are resistant to lodging.

**Supplementary Information:**

The online version contains supplementary material available at 10.1186/s13007-023-01075-1.

## Background

Field pea (*Pisum sativum* L.) is a versatile pulse crop with significant potential benefits in human nutrition and sustainable cropping systems, particularly through nitrogen fixation and soil improvement [1]. As with other grain legumes, field pea is a rich source of protein, fibre, vitamins, and complex carbohydrates, making it an excellent addition to the human diet [2]. Field pea also plays a critical role in sustainable farming systems, improving soil fertility through nitrogen fixation, adding organic matter, promoting soil nutrient cycling and water infiltration, and breaking disease cycles of cereal crops. These soil-improving qualities provide multiple benefits to the agroecosystem, contributing to a more sustainable and resilient farming system [3].

Despite these benefits, field pea farming encounters significant agronomic challenges, with one prominent issue being stem lodging [4]. Stem lodging refers to a condition in which the stems of the crop bend near the soil surface due to inadequate support from the lower internodes, causing the collapse of the canopy [5]. Stem lodging is a major constraint in field pea production, as it results in a humid microclimate suitable for fungal diseases and reduces photosynthetic efficiency, resulting in lower yield potential and increasing the cost and difficulty of harvest [6–8]. Field peas tend to have weak stems and depend on tendrils for support, which ultimately do not prevent the crop from lodging [9]. A major goal of field pea breeding is to increase stem strength [10] and improve our understanding of the mechanical properties underlying lodging susceptibility [11]. Therefore, it is critical to obtain reliable and repeatable stem strength measurements for field pea breeding.

A standard method used to measure the stem strength of field pea is 3-point bending with a mechanical testing device, such as a uniaxial compression tester, which is measured on an excised stem from a mature dry plant [12, 13]. In this method, a mechanical load is applied laterally on the stem between two points and the deviation from the original position of the sample (flexion) and load are recorded at the breaking point [12, 13]. Another method used to characterise stem strength involves measuring the cutting work or the cutting work per unit area at the stem cross-section with the help of a Dynstat apparatus [14]. Stem buckling is an additional method of estimating stem strength that has recently been explored in peas [15]. However, these mechanical assessment methods require breaking or cutting stems, which is destructive to the plant and makes it impossible to repeatedly assess the stem strength when it is alive.

An alternative method for measuring stem strength involves measuring compressed stem thickness (CST) as an indicator of strength [16]. This is achieved by applying firm finger pressure to the side of the stem and measuring the thickness of the compressed stem between the jaws of a Vernier calliper [16]. Results from this work show that there was a highly significant linear regression (${r}^{2}$= 0.92) between CST and the square-root transformed load at the breaking point in the 3-point bending test [16]. CST is related to stem wall thickness and stems should become stronger when the walls become thicker, but there is a trade-off between weight per unit length and stiffness [17]. CST may be measured more objectively by applying a standardised force laterally to the stem against a solid surface and measuring the displacement from the full diameter of the stem [15]. Due to time and resource limitations, most previous work on measuring stem strength has been restricted to one internode, such as internode 3 [13, 15, 16, 18]. Also, CST requires the stem to be cut and dried before measurement, which is destructive and may not accurately reflect in vivo conditions.

In this paper, we propose a novel method for assessing stem wall thickness on living plants using optical coherence tomography (OCT) [19, 20]. OCT is a rapid, high-resolution optical imaging technique that allows for depth-sectioning in plant tissue, enabling the visualisation of stem structures in two dimensions (2D) or three dimensions (3D). Although OCT has mainly been demonstrated in biomedical applications, such as ophthalmology [21, 22], oncology [23–26] and cardiology [27, 28], several preliminary studies have shown that OCT can also provide non-destructive measurements of plants in agricultural applications [29–31]. Given that OCT can penetrate turbid materials to depths up to several millimetres, it is well-suited to measuring stem wall thickness in living plants. Moreover, this non-invasive approach does not require contact or compression of the stem, allowing for the assessment of traits related to lodging at early stages of growth without compromising the plant’s development cycle or its ability to produce seeds for selection.

To demonstrate the capability of OCT to measure stem wall thickness, we conducted in vivo imaging on two cultivars of field pea, Dunwa and Kaspa. The refractive index of the stem tissue was measured by calculating the ratio between the optical thickness and physical thickness of the same stem tissue in OCT images. The in vivo thickness measurements were adjusted for the refractive index of the stem tissue. To validate the accuracy of our in vivo measurements, we extracted the imaged stems from the plants and aligned the direction of stem wall thickness with one of the lateral axes of the OCT system. By precisely calibrating the distance measurement in the lateral axes of the OCT system, we were able to accurately measure the ex vivo stem wall thickness using OCT. This ex vivo measurement then served as a reliable reference for the in vivo measurements we obtained, further ensuring their accuracy and validity. Additionally, we performed in vivo OCT imaging on live field pea plants to measure stem wall thickness and stem width at various internode positions. We believe that our method can be used to provide valuable insights for selection against stem lodging in field pea breeding programs.

## Materials and methods

### Plant samples

**Fig. 1:**
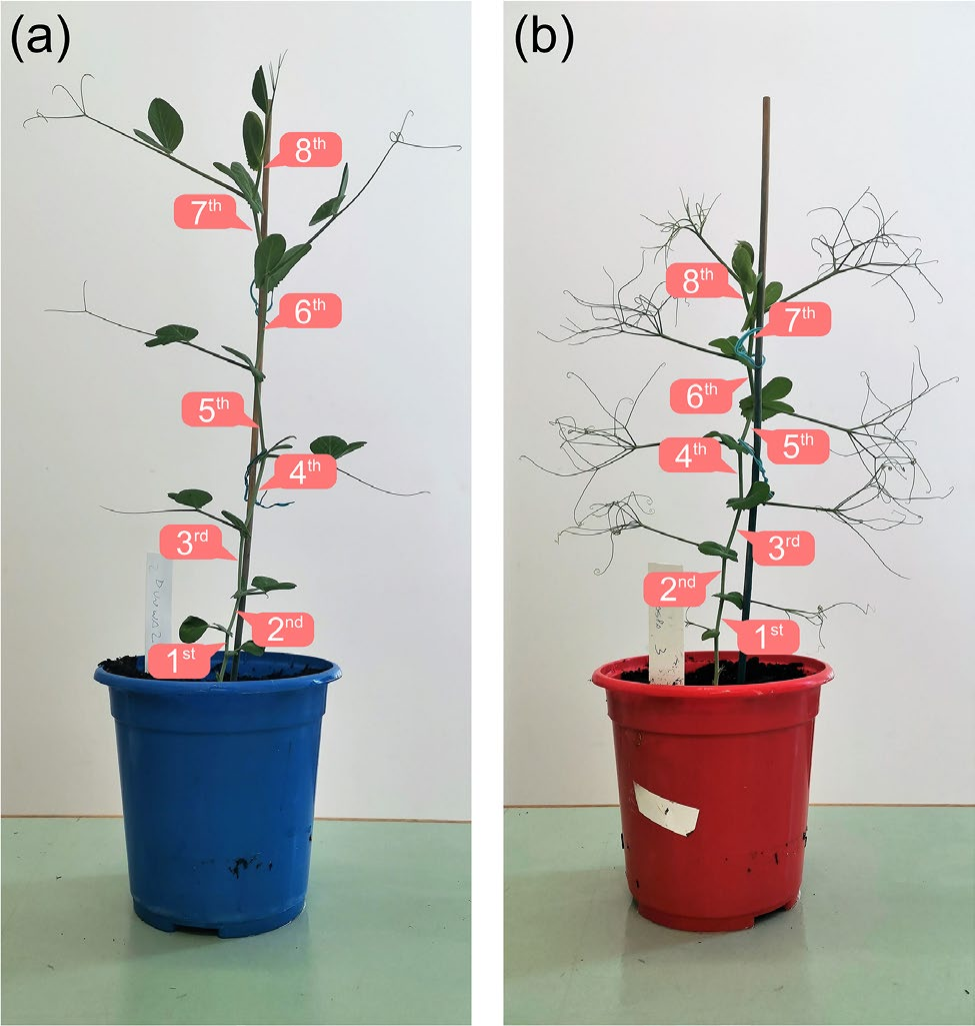
Examples of (**a**) Dunwa and (**b**) Kaspa plants used in the experiments. The 1^st^ to 8^th^ internode positions are labelled for each plant

Two Australian-bred field pea cultivars were used in this study: Dunwa, a traditional trailing growth type and Kaspa, a semi-leafless growth type [16]. Replicates of both cultivars were sown directly into 1 L plastic pots during autumn, using commercially available potting mix with slow-release fertiliser. The pots were transferred to a glasshouse at The University of Western Australia (UWA) Field Station, Shenton Park, Western Australia, and plants were grown under glasshouse conditions with automated watering and controlled temperature. Upon emergence, seedlings were supported by stakes to allow for upright development of the initial portion of the stems. After the plants reached the 8^th^ to 10^th^ node growth stage, which typically occurred around 4-6 weeks after emergence, they were transferred to the Department of Electrical, Electronic & Computer Engineering at UWA in Crawley, Western Australia. The transfer process was completed within 30 min. Upon arrival, the plants were placed under fluorescent tube lighting for 12 h each day at room temperature (22-23 °C), and were kept adequately watered to avoid moisture stress. The plant stems were measured within three days of transfer. In the first experiment, in which in vivo and ex vivo measurements of stem wall thickness were compared, 10 plants of each cultivar were measured approximately four weeks after emergence, when they reached the 8^th^ node growth stage. In the second experiment, in which in vivo stem wall thickness was measured at various internode positions, seven plants of each cultivar were assessed approximately six weeks after emergence, when they reached the 10^th^ node growth stage. Figure 1(a) and 1(b) show examples of Dunwa and Kaspa plants, respectively, approximately four weeks after emergence. The internode positions are labelled from the 1^st^ to the 8^th^ internode.

### Imaging system

**Fig. 2:**
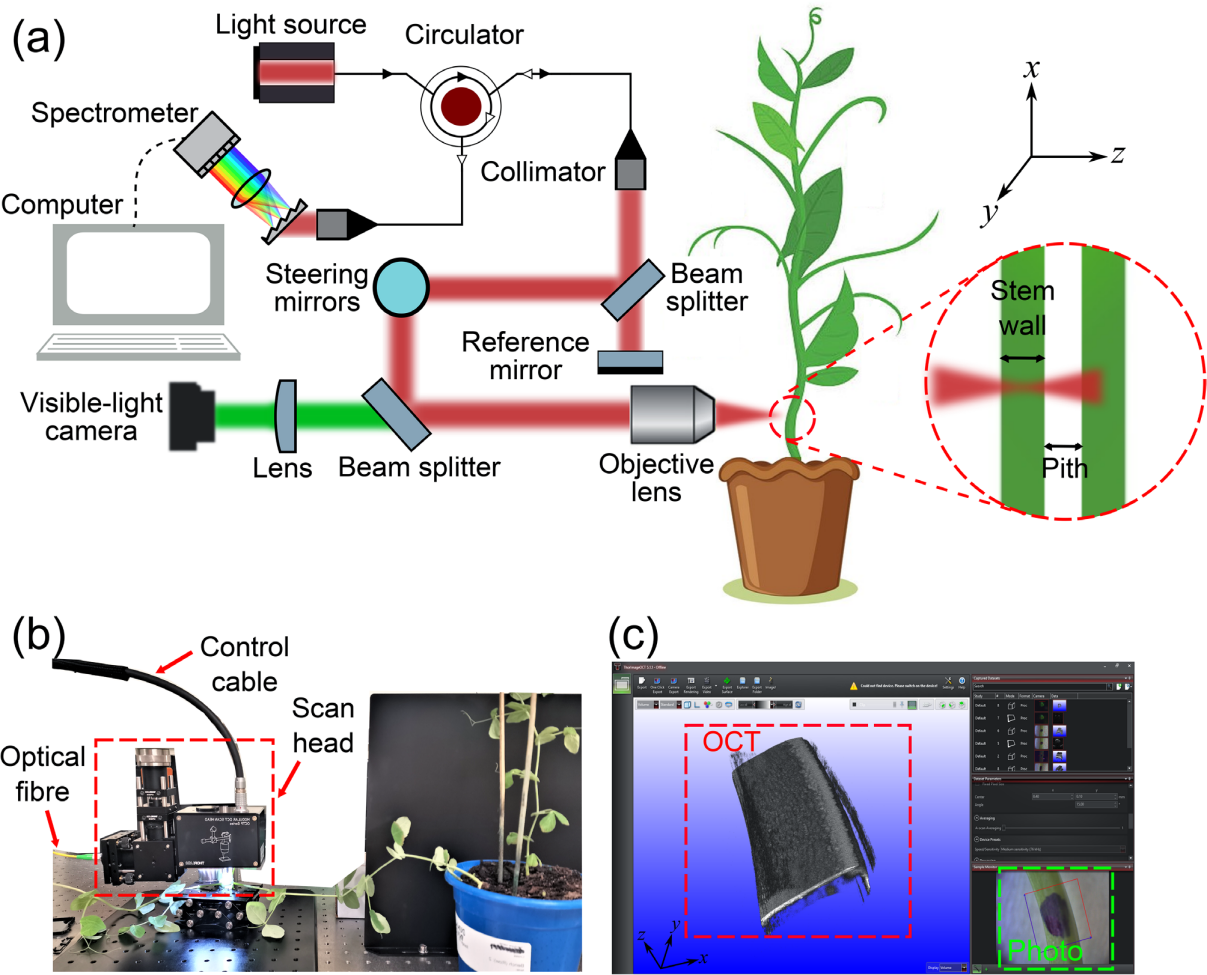
(**a**) Schematic diagram of OCT for in vivo stem wall thickness measurements. (**b**) Photograph of the experimental setup. (**c**) Software interface for visualisation and measurement

The imaging system used in this study is a spectral-domain OCT system (Telesto III OCTP-1300, Thorlabs) with a superluminescent diode source (1300 nm central wavelength, full-width-at-half-maximum (FWHM) bandwidth of 220 nm). Figure 2(a) shows a schematic diagram of the experimental setup.

The light beam from the source propagates through optical fibres and enters an optical circulator. The output beam from the circulator is expanded into a collimated beam in free space before being split into two beams. One of the beams travels directly to a reference mirror and is reflected along the same path. The other beam is directed onto two galvanometer scanning mirrors, which scan the beam in orthogonal directions in the object plane. An objective lens (OCTLK3, Thorlabs) focuses the scanned beam onto the sample, allowing some of the light to reach the inner surface of the stem wall. The light back-scattered from the sample travels back along the same path as the incoming beam and meets the beam reflected from the reference mirror at the beam splitter. These two beams then propagate through the circulator and interfere at the spectrometer. The spectral interferogram is recorded by a computer and converted to depth-resolved OCT intensities via an inverse Fourier transform [32].

The measured axial ($z$) and lateral ($xy$) resolution (FWHM) of the OCT imaging system are 5.5 μm (in air) and 7.8 μm, respectively, and the maximum field of view (in air) is 9.4 × 9.4 × 3.5 mm^3^. In addition to OCT, a visible light photograph of the sample is acquired using a camera and a second beam splitter. Figure 2(b) shows a photograph of the experimental setup for in vivo stem wall thickness measurements. The scan head contains the collimator, the beam splitters, the reference mirror, the scanning mirrors, the objective lens, and the visible light camera. Once the data are acquired and processed, 2D and 3D images of the sample can be viewed using the OCT software (ThorImage 5.5, Thorlabs), as shown in Fig. 2(c). The brightness in the acquired images corresponds to the signal-to-noise ratio (intensity) of the OCT intensity, which is adjusted to 20-40 dB for optimal contrast at the stem wall surfaces. The dimensions of the sample can be measured using this software. Any field of view in the photograph can be selected, and the beam is scanned in two orthogonal directions at the same region of the sample to generate OCT images. In the experiments, we set the lateral field of view to be 4 × 4 mm^2^, which is sufficient to image the entire stem cross sections. The OCT acquisition rate was set to be 76 kHz and resulting images comprised 1000 pixels in each of the two lateral directions. Two-dimensional, cross-sectional OCT images (B-scans) were acquired along the fast-scanning axis ($xz$-plane), while OCT volumes were acquired by combining a series of B-scans along the slow-scanning axis ($y$-axis). The acquisition time is 13 milliseconds for each Bscan and 13 s for each OCT volume.

The distance measurement in the axial direction ($z$) is achieved by counting the pixels in the OCT images, where each pixel in the $z$-direction corresponds to 3.4 μm in air, determined by the pixel number and resolution of the spectrometer. To ensure accurate distance measurements in both lateral directions, the OCT system was calibrated using a 3-hole metal plate sample (M0096-70-0374, Thorlabs) specifically designed to calibrate the Telesto OCT system, and a positive concentric square target (R3L3S3P, Thorlabs) for validating the calibration.

### Imaging protocols

**Fig. 3:**
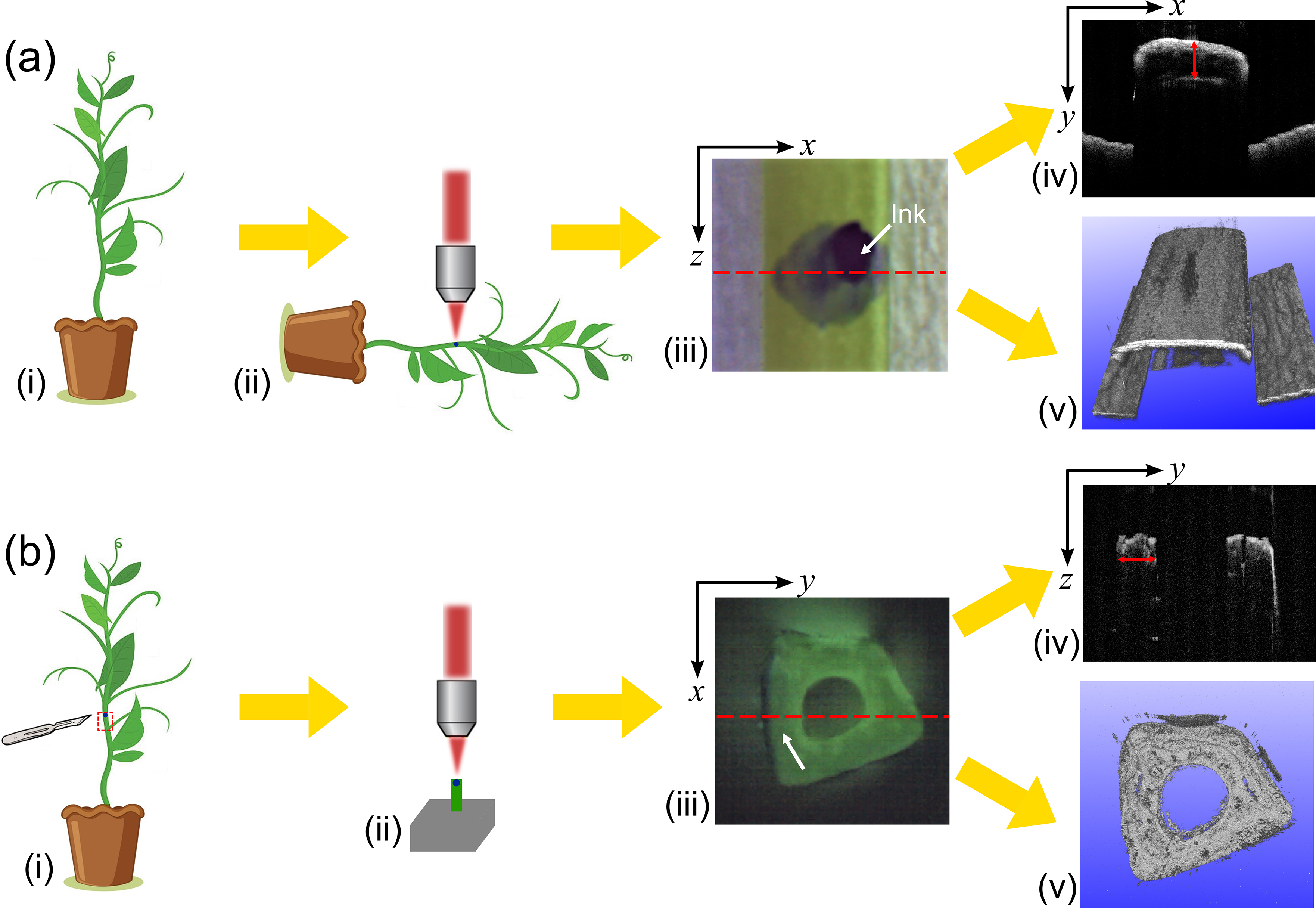
Protocols for (**a**) in vivo and (**b**) ex vivo measurement of the stem wall thickness using OCT. (**a**): (i)-(v) illustrate the steps required to generate OCT images for in vivo stem wall thickness, and (**b**): (i)-(v) illustrate the steps required to generate OCT images for ex vivo stem wall thickness. The example photographs and OCT images (**a**): (iii)-(v) and (**b**): (iii)-(v), respectively, were acquired from the 7^th^ internode of a Dunwa plant

We developed a protocol for in vivo measurement of stem wall thickness using OCT. As shown in Fig. 3(a), we first prepared a plant (i) and positioned it so that the stem lay on top of a stage under the objective lens of the OCT system (ii). We adjusted the height of the scan head to focus the beam approximately 500 μm below the sample surface. To mark the position to be measured, we inked the stem with a marker pen (iii). Due to the minimal amount of ink used, the ink on the stem tissue is negligible and not noticeable in the OCT scans. Using the acquisition software, we acquired a B-scan (iv) at the stem location marked by the red dashed line across the inked position in (iii). In the B-scan (iv), the outer surface of the stem appears as a bright line at the top of the image. The inner surface of the stem wall appears as another line with lower OCT intensity, due to optical attenuation in the stem tissue. The red double-ended arrow in (iv) represents the optical distance between the inner and outer surfaces of the stem wall. This optical distance can be measured by counting the pixels between the two surfaces and converting the pixel number to a distance measurement (3.4 μm/pixel) using the ruler function in the OCT software. The stem wall thickness, $T$, is the physical distance between these two surfaces which is given by:

1 $$T=\frac{{T}_{opt}}{{n}_{stem}},$$

where ${T}_{opt}$ is the optical path length of the light beam through the stem wall (optical thickness) and ${n}_{stem}$ is the refractive index of the stem tissue. Using Eq. (1), the stem wall thickness can be calculated from the measurement of ${T}_{opt}$, if ${n}_{stem}$ is known. We use OCT system to measure the optical thickness and the physical thickness of the same tissue sample and calculated the ratio to determine ${n}_{stem}$. Details of this measurement are described in the next section. An OCT volume can also be generated (v), enabling the 3D stem structure to be visualised. The imaging field of view of (v) matches that of the photograph (iii).

To validate that in vivo OCT imaging can accurately measure the stem wall thickness of field pea, we also performed ex vivo stem wall thickness measurements and compared them with the in vivo measurements. In the ex vivo measurements, the stem tissue was excised so the stem wall could be clearly identified (Fig. 3(b)). As the OCT system was precisely calibrated in both lateral directions, the stem wall thickness can be accurately measured by imaging the stem cross section ($xy$-plane) by OCT. The ex vivo measurement protocol is displayed in Fig. 3(b). We first cut a 1 cm long slice of stem from the same plant location assessed in the in vivo measurement, with one end cut at the position marked by ink (i). We then placed this slice of stem on top of a flat stage to face the stem cross-section ($xy$-plane) towards the objective lens and adjusted the OCT beam to focus on the top end of the stem (ii). During this procedure, the excised stem was immediately transferred to the stage for imaging to avoid errors caused by the shrinkage of the stem due to water loss. The OCT imaging range was set such that the entire cross section of the stem ($xy$-plane) was imaged, as shown in (iii) and (v). From the photograph (iii), it is evident that the inside of the stem has a hollow space (typical for stems beyond the 3^rd^ internode position of the plant, details shown in Results). The inked side of the stem is marked by a white arrow in this photograph. A B-scan was acquired for the location marked by the red dashed line in (iii), as displayed in (iv). The inner and outer surfaces of the stem walls can be distinguished in this OCT B-scan. The red double-ended arrow represents the distance between the inner and outer surfaces of the inked stem wall. This distance can be measured using the software’s ruler tool and serves as a reference for the in vivo measurement. Again, OCT volumes can be acquired for the excised stem piece for visualisation of the stem structure (v). The field of view in (v) is the same as in (iii).

### Refractive index measurement

**Fig. 4:**
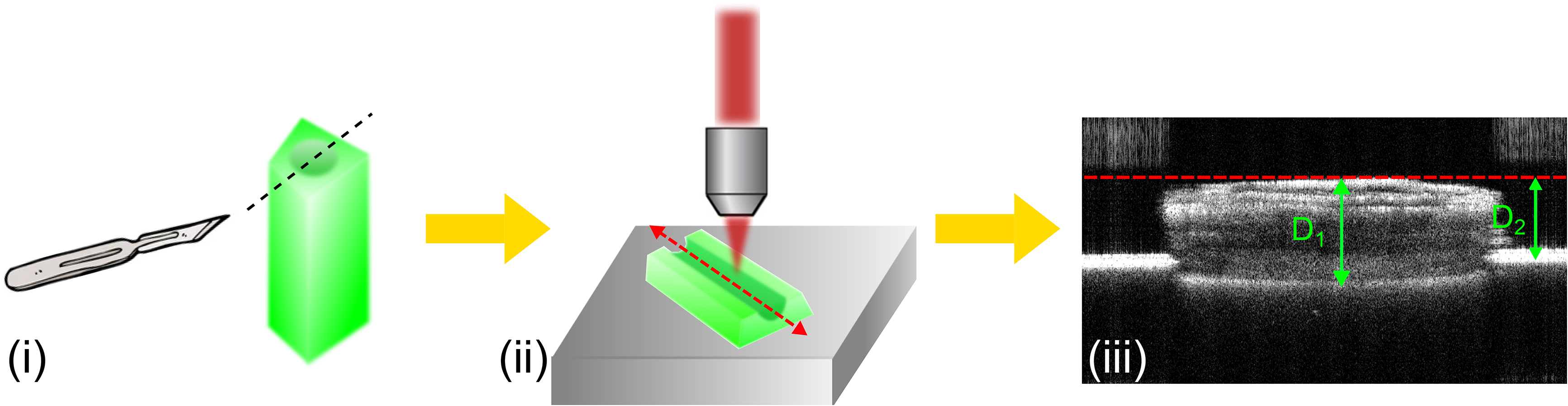
Procedure used for refractive index measurements. (**i**) An excised stem is cut in half along the black dashed line. (**ii**) One half of the stem sample is imaged using OCT. The OCT scan direction is indicated by the red dashed line. (**iii**) A B-scan acquired along the red dashed line in (ii). The physical thickness (D_2_) and the optical thickness (D_1_) of the stem tissue are measured to calculate the refractive index

To provide accurate in vivo measurement of the stem wall thickness, the refractive index of the stem tissue needs to be characterised. In our work, we used OCT measurements to determine the refractive index of the stem tissue, as illustrated in Fig. 4. This method has been described in detail previously [33, 34]. We first excised a 1 cm long stem from a live plant and cut the stem in half along the stem axis (i). One half of the stem was placed on top of a metal stage and the OCT beam was focused and scanned along the stem axis, as marked by the red dashed line in (ii). We then acquired a B-scan for the marked stem location (iii). In this B-scan, we measured the distance between the top and bottom surfaces of the stem sample (D_1_) and the distance between the top surface of the sample and the metal stage (D_2_). As the stem sample was placed closely against the metal stage, the bottom surface of the sample and the stage surface were at the same location. In the OCT image (iii), D_2_ represents the physical thickness and D_1_ represents the optical thickness of the stem tissue. The refractive index of the stem tissue ${n}_{stem}$ is given as:

2 $${n}_{stem}=\frac{{D}_{1}}{{D}_{2}}.$$

In the experiment, we measured the refractive index as 1.33 ± 0.01 for three Dunwa stems and three Kaspa stems. In all the in vivo measurements of stem wall thickness presented, we set the refractive index of the stem tissue as ${n}_{stem}=1.33$.

Note that the refractive index of the stem tissue can cause the incident light beam to refract when it passes from air into the tissue, particularly if the stem is positioned obliquely to the beam. Consequently, there is a possibility of a slight deviation in the measured stem wall thickness from its actual physical thickness. However, it is important to emphasise that the oblique angles encountered in most of our in vivo OCT measurements are relatively small. As a result, the impact of light beam refraction on the accuracy of the stem wall thickness measurement is negligible. For a more comprehensive understanding of this effect and its implications, a detailed analysis is provided in Supplementary S1.

## Results

### Ex vivo stem structure imaging

**Fig. 5:**
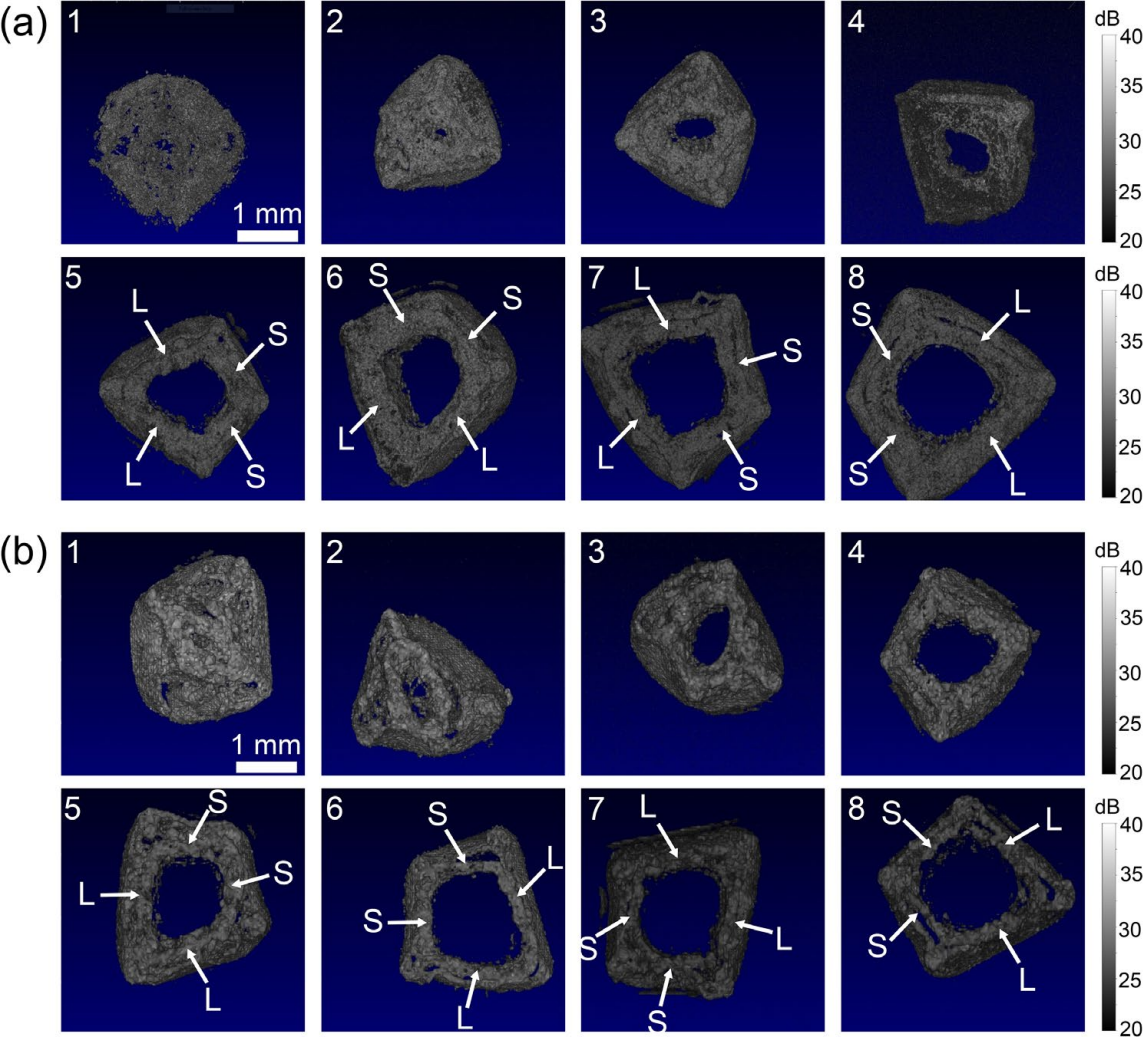
Ex vivo 3D OCT images of field pea cultivar (**a**) Dunwa and (**b**) Kaspa cross sections from the 1^st^ to 8^th^ internode positions. L, long side. S, short side. The grayscale colour bars represent OCT intensity. All the images in this figure have the same scale bar

In this project, we utilised OCT to visualise the ex vivo stem structure by imaging stem cross sections ($xy$-plane) at different internode positions. Figure 5 shows representative ex vivo OCT volumes of stem cross-sections from the 1^st^ to the 8^th^ internode positions on 4-week-old Dunwa (a) and Kaspa (b) plants. Each ex vivo image has a lateral field of view of 4 × 4 mm^2^. As observed in Fig. 5, field pea stems exhibit a distinctive hollow structure, referred to as the pith. This anatomical characteristic is easily observed by the naked eye or with the aid of a microscope [14]. It can be seen in Fig. 5 that the pith is not fully developed until the third internode for both cultivars, with the diameter of the pith continuing to increase until the 6^th^ or 7^th^ internode. From the third internode onwards, the internal stem wall can be clearly identified, enabling in vivo measurement of stem wall thickness beyond this internode position. It can be observed that the cross section of the stem typically resembles a kite shape with two relatively long sides (L) and two relatively short sides (S), which can be distinguished from the 5^th^ internode onwards.

### Validation of in vivo measurement

**Fig. 6:**
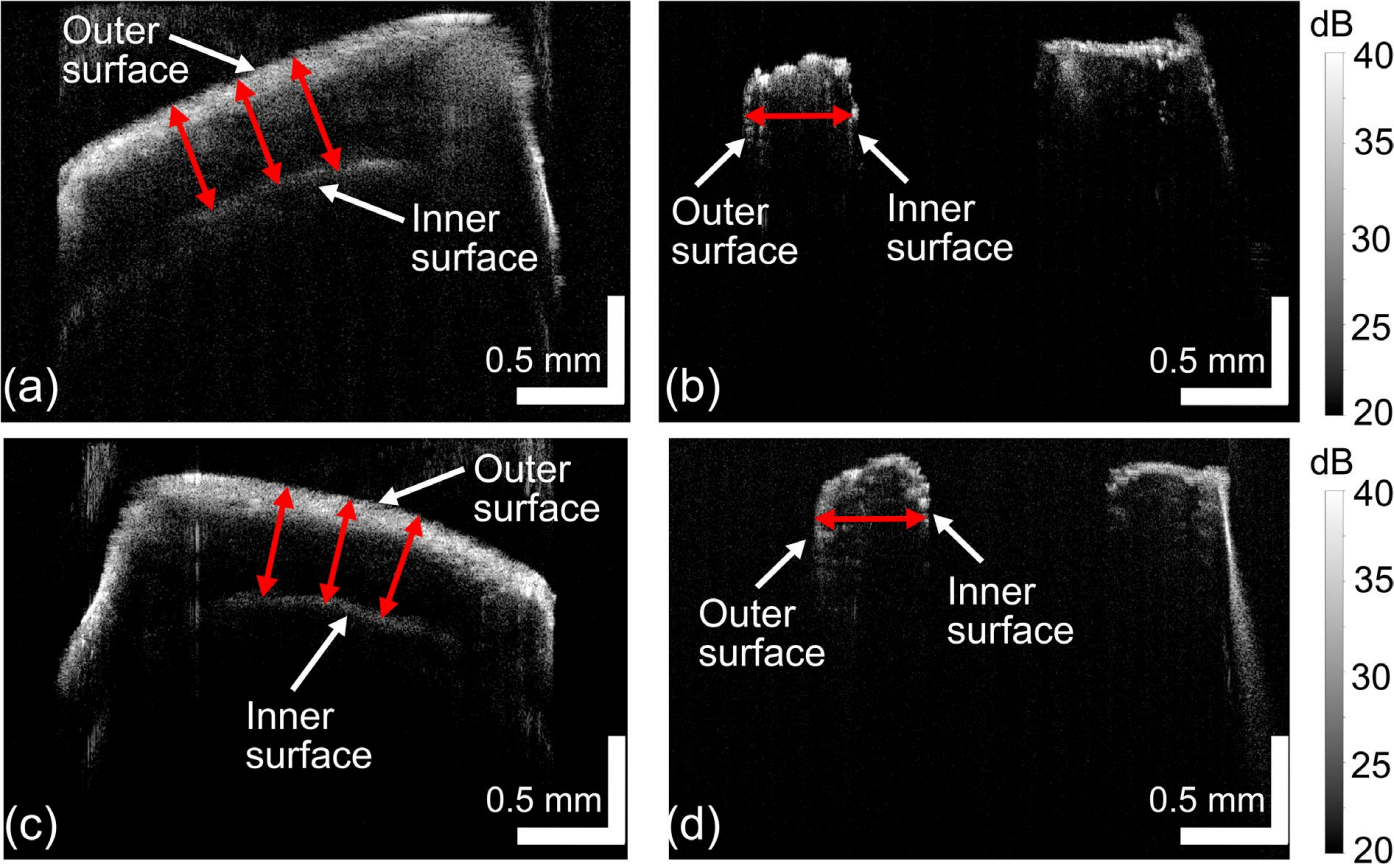
Representative (**a**) in vivo and (**b**) ex vivo measurements of **a** Dunwa stem at the 7^th^ internode position. Representative (**c**) in vivo and (**d**) ex vivo measurements of a Kaspa stem at the 7^th^ internode position. The grayscale colour bars represent OCT intensity

To validate the accuracy of OCT in measuring in vivo stem wall thickness, we conducted both in vivo and ex vivo measurements on 10 plants each of Dunwa and Kaspa at four weeks of age. Figure 6 shows examples of both measurements for Dunwa and Kaspa. Figures 6(a) and 6(c) show in vivo B-scans of the stem wall thickness at the 7^th^ internode position for Dunwa and Kaspa, respectively. In these images, the outer surface of the stem wall appears as a bright line at the top of the image due to the high refractive index difference between the stem tissue and air. The inner surface of the stem wall appears dimmer as the optical beam is attenuated and scattered while propagating through the stem wall. To ensure accurate in vivo measurement of stem wall thickness, we performed three measurements at various locations across the stem width, as indicated by the red arrows in the figures, and calculated the mean thickness. Following imaging, we marked the B-scan location for the in vivo measurement and excised a 1-cm long stem slice from that location. Figure 6(b) and 6(d) show the corresponding ex vivo OCT B-scans of the stem cross section at the marked location. The width of the stem wall was directly measured in these B-scans, as indicated by the red arrows in (b) and (d).

**Fig. 7:**
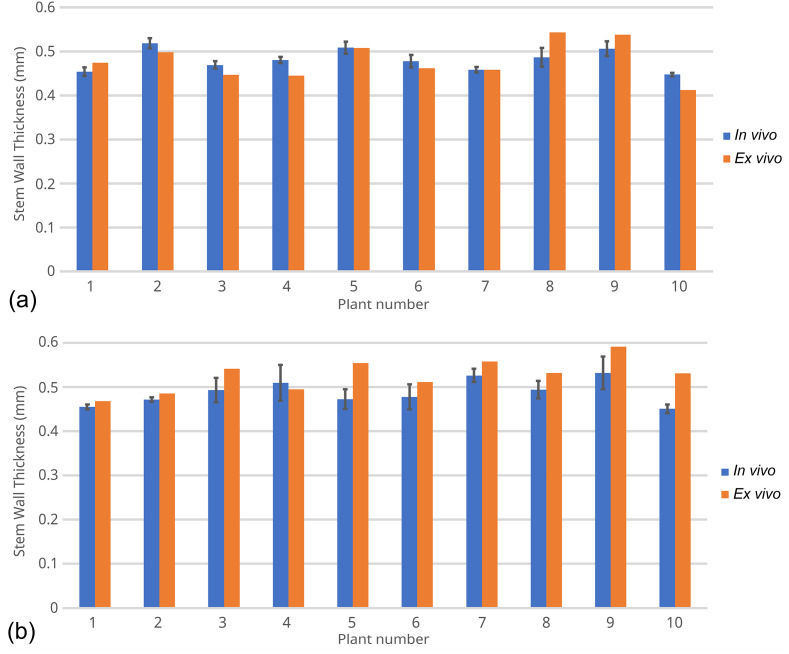
In vivo and ex vivo measurements of 10 plants each of field pea cultivars (**a**) Dunwa and (**b**) Kaspa at the 7^th^ internode position

To compare the in vivo and ex vivo measurements of stem wall thickness, we performed in vivo OCT on 10 plants each of Dunwa and Kaspa at the 7^th^ internode position, and then excised the stem from the same location for ex vivo measurement. We selected the 7^th^ internode position for measurement, as stem structure was fully developed at this position so the stem wall surfaces could be easily identified in OCT. The results for these 20 plants are summarised in Fig. 7. The blue columns in Fig. 7 represent the mean in vivo stem wall thickness, while the height of each column is the mean value of the three in vivo measurements, and the error bar denotes the standard deviation of these measurements. The orange columns in Fig. 7 represent the ex vivo measurements of the excised stems. The percent error between the in vivo and ex vivo measurements can be expressed as:

3 $$\delta = \frac{{{T_i} - {T_e}}}{{{T_e}}} \cdot 100\% ,$$

where ${T}_{i},{T}_{e}$ are the mean stem wall thicknesses from the in vivo and ex vivo measurements, respectively. In Fig. 7(a), the mean stem wall thickness across the 10 Dunwa samples is 0.481 mm for in vivo and 0.479 mm for ex vivo measurements, resulting in a percent error ${\delta }_{Dunwa}$ = 0.9% using Eq. (3), with a standard deviation of 5.9%. Similarly, in Fig. 7(b), the mean stem wall thickness across the 10 Kaspa samples is 0.488 mm for in vivo and 0.527 mm for ex vivo measurements, with a percent error ${\delta }_{Kaspa}$ = − 7.1% and a standard deviation of 5.3%. The mean percent error between in vivo and ex vivo measurements for these 20 plants is − 3.1%. The raw data of this experiment is included in Tables S1 and S2 in the Supplementary. The discrepancy observed between the in vivo and ex vivo measurements is attributed to the minor structural distortion that occurred when excising the stem tissue with a scalpel blade, as well as any variation in refractive index of the measured stem tissue from the preset value ${n}_{stem}=1.33$.

### In vivo measurement at different internode positions

**Fig. 8:**
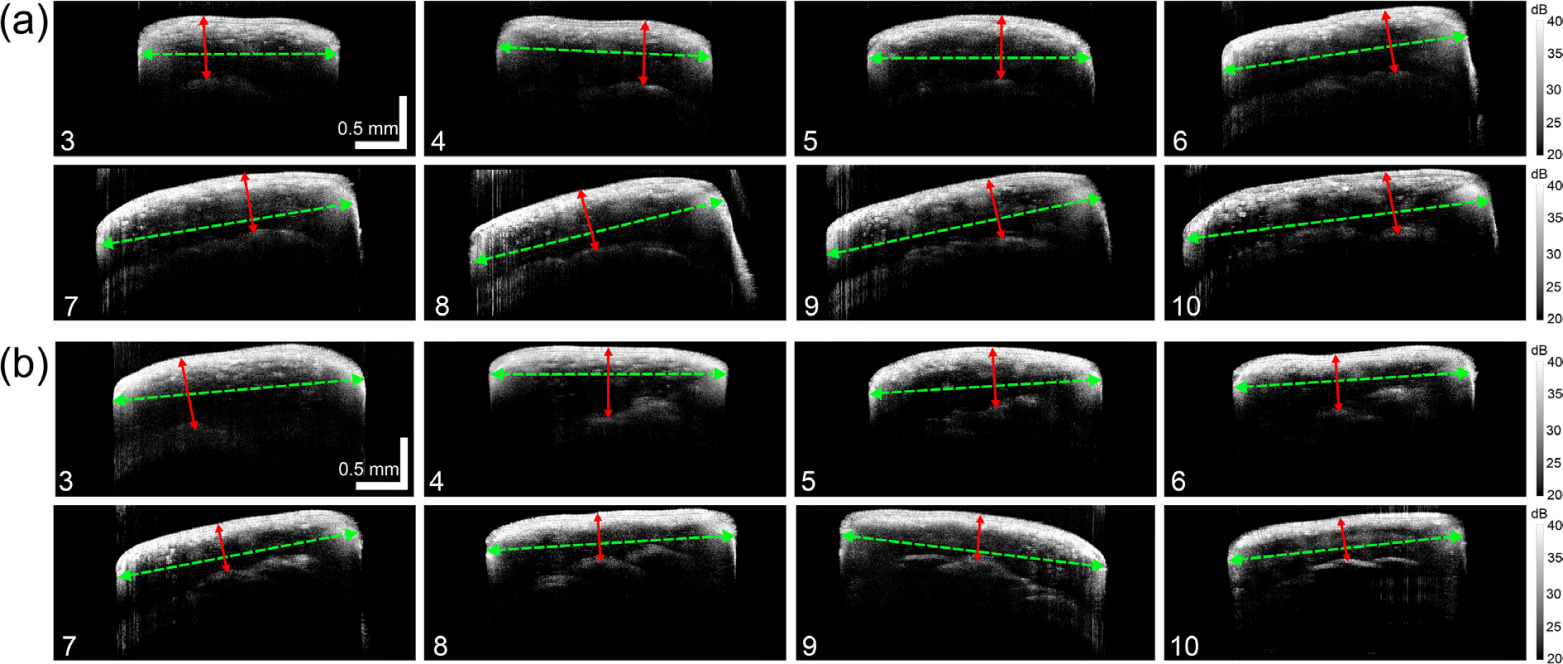
Examples of in vivo measurements of (**a**) a Dunwa plant and (**b**) a Kaspa plant across the long side of the stem from the 3^rd^ to 10^th^ internode positions. The grayscale colour bars represent OCT intensity. All the images in this figure have the same scale bar

To demonstrate in vivo OCT measurement of stem thickness, we imaged seven Dunwa and seven Kaspa plants when they were six weeks old and measured the stem wall thickness and the stem width at internode positions 3 to 10. As shown in Fig. 5, the 1^st^ and 2^nd^ internode positions lack an obvious hollow structure, making it challenging to measure stem wall. Figure 8(a) and 8(b) illustrate examples of in vivo OCT Bscans from the 3^rd^ to 10^th^ internode positions across one of the long sides of the stem for a Dunwa and a Kaspa plant, respectively. The stem wall thickness was determined as the distance between the outer and inner surfaces of the stem wall, marked by the red double-ended arrow in each image. A comparison between these images revealed that the stem wall thickness is relatively uniform across internode positions for Dunwa while it gradually reduces along the internode positions for Kaspa. Furthermore, we observed that the measured stem width (indicated by the green dashed line with double-ended arrows) on the long side is small at lower internode positions and gradually increases at higher internode positions for Dunwa, while changes in stem width are relatively small across internode positions for Kaspa.

**Fig. 9:**
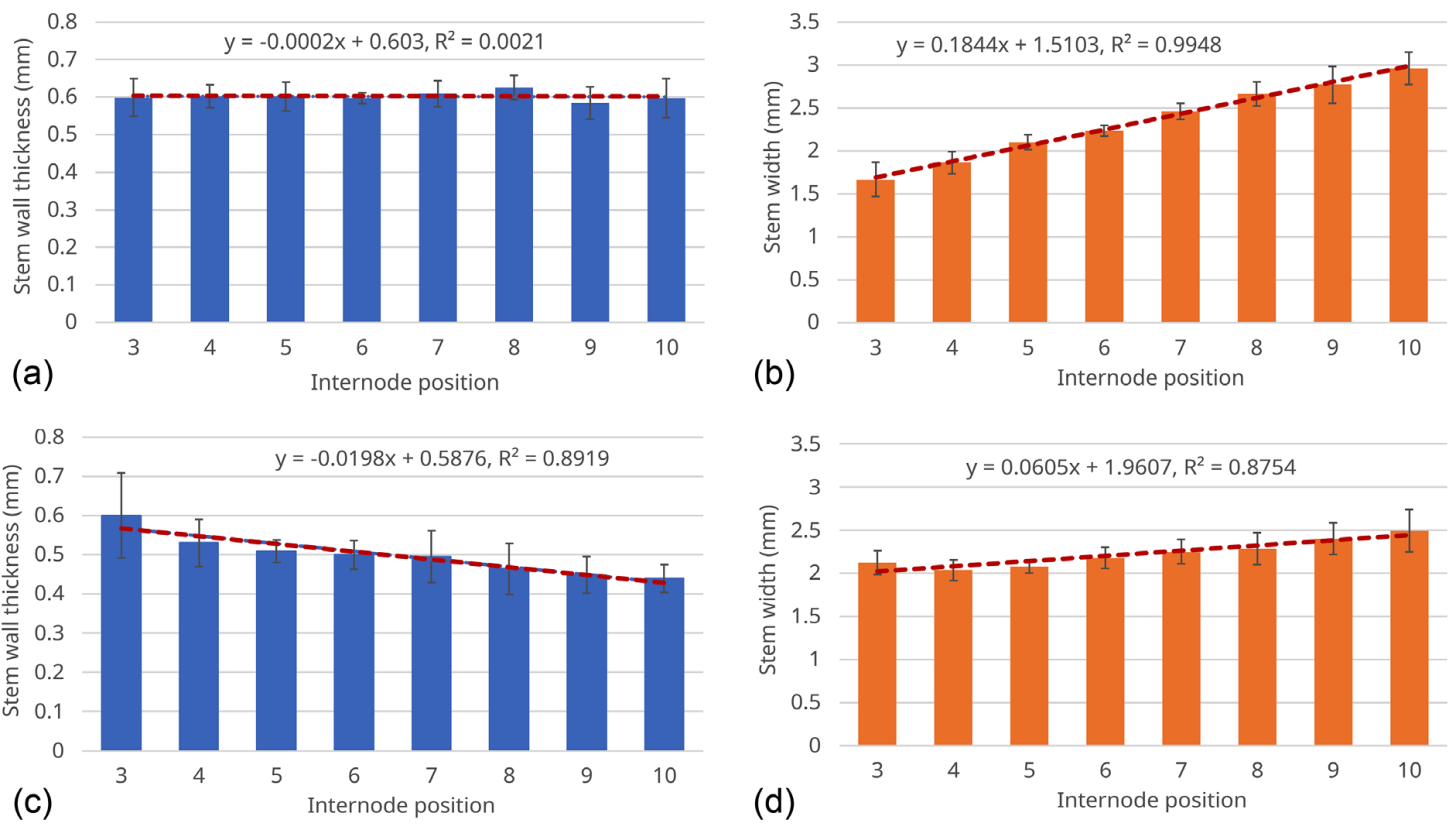
(**a**) in vivo stem wall thickness and (**b**) stem width measurements at different internode positions for seven plants of Dunwa. (**c**) in vivo stem wall thickness and (**d**) stem width measurements at different internode positions for seven plants of Kaspa. The error bars are the standard deviations calculated from the seven plants at each internode position

The measurements of stem wall thickness and stem width for the 3^rd^ to 10^th^ internode positions of the seven Dunwa and seven Kaspa plants are summarised in Fig. 9. Each blue column in Fig. 9(a) represents the mean stem wall thickness measured at the same internode position across the seven Dunwa plants and the error bar indicates the standard deviation. To assess the change in stem wall thickness at different internode positions, we performed linear regression to the mean stem wall thickness, as shown by the red dashed line in Fig. 9(a). The line of regression has the expression:

4 $${y}_{T\_Dunwa}=-0.0002x+0.6030,$$

where $x$ represents the internode position and ${y}_{T\_Dunwa}$ is the stem wall thickness at different internode positions for the Dunwa plants. The calculated coefficient of determination is ${R}^{2}=0.0021$. The small coefficient of − 0.0002 mm/node in Eq. (4) is not significant ($\text{P}=0.9143$) and the small ${R}^{2}$ value confirms the nonsignificant linear regression, corresponding to relatively uniform stem wall thickness across the different internode positions of the Dunwa plants. The standard error of this linear regression is 0.0128 mm, indicating a reliable fitting of this linear regression to the stem wall thickness measurements.

The stem width measurements of the seven Dunwa plants from the 3^rd^ to 10^th^ internode positions are presented as the orange columns in Fig. 9(b). It is evident that the stem width increases significantly along the internode positions. The red dashed line represents the linear regression for the mean values of the stem width across the seven plants and the error bars represent standard deviation. The line of regression of the stem width along the internode positions for the Dunwa plants is expressed as:

5 $${y}_{W\_Dunwa}=0.1844x+1.5103.$$

The coefficient of determination of this linear regression is given as ${R}^{2}=0.9948$ and its standard error is 0.0353 mm. The slope of the regression is 0.1844 mm/node, which is significantly different from zero ($\text{P}<0.0001$).

Figure 9(c) presents the in vivo stem wall thickness measurements of the seven Kaspa plants. Each column is given as the mean value across the seven plants and the error bar is the standard deviation. We fitted the mean stem wall thickness with a line of regression given as:

6 $${y}_{T\_Kaspa}=-0.0198x+0.5876.$$

The coefficient of determination is given as ${R}^{2}=0.8919$. The coefficient in Eq. (6) is significantly negative (− 0.0198 mm/node, $\text{P}=0.0004$), indicating that the stem wall thickness decreases as internode position increases in Kaspa. The standard error of this linear regression is 0.0183 mm. According to the in vivo measurements and the linear regression, it is shown that the Kaspa plants tend to have thicker stem walls at the lower internode positions and thinner stem walls at the higher internode positions.

Figure 9(d) presents the stem width measurements of the seven Kaspa plants from the 3^rd^ to 10^th^ internode positions (orange columns). The red dashed line is the linear regression fitted for the mean value of the stem width and the error bars are given by the standard deviations across the seven plants. The line of regression of the stem width across different internode positions is given as:

7 $${y}_{W\_Kaspa}=0.0605x+1.9607.$$

The coefficient of determination of this linear regression is ${R}^{2}=0.8754$ and its standard error is 0.0604 mm, with $\text{P}=0.0006$. Comparing Fig. 9(b) and 9(d), it is observed that the stem width increases linearly as internode position increases across the range of 2.0-2.5 mm for Kaspa and 1.5-3.0 mm for Dunwa, i.e., the rate of increase in stem width is three times higher in Dunwa than in Kaspa. Overall, the trends in the stem characteristics (stem wall thickness and stem width) in this set of samples are different for the two cultivars (Fig. 9).

It is important to acknowledge that the linear regressions in Fig. 9 serves as a simple model aimed at explaining the overall trends of the stem wall thickness and stem width with respect to the internode position. However, these linear regressions may not be the most optimal model. To build a more accurate and comprehensive model, a larger dataset encompassing more samples is needed. By fitting a large dataset to a more sophisticated and nonlinear function, we will be able to obtain a more refined understanding of the relationship between stem wall thickness and stem width with respect to the internode position.

## Discussion

In previous studies, it has been reported that the thickness of the stem walls of field peas (as estimated by compressed stem thickness) is a more significant determinant of stem strength than the outer diameter [16] which conforms to theoretical expectations of the strength of a cylinder [17]. In this paper, we introduce a new approach for in vivo imaging of stem wall thickness in field pea stems with achieved accuracy of − 3.1% compared to destructive ex vivo measurements, and which contributed to our understanding of stem strength and lodging resistance in living plants. Compared to traditional methods that require the stems to be cut and dried, OCT offers the benefits of in vivo measurement and rapid data acquisition on living tissue, and therefore potentially provides more meaningful assessment of living plant traits related to lodging resistance.

In this study, we utilised OCT to image two field pea cultivars, Dunwa and Kaspa, and to measure stem wall thickness and stem width in vivo. Figure 9 shows distinct trends in stem wall thickness and stem width along internode positions for these two cultivars, potentially indicating different responses to lodging. This suggests that the internal stem morphology and, therefore, the stem strength may vary between these two field pea cultivars. In previous ex vivo studies, time and resources restricted measurement of stem wall thickness to the third internode [13, 15, 16, 18]. OCT reveals that Kaspa and Dunwa have similar stem wall thickness at the third internode, but Dunwa has thicker stem walls than Kaspa at higher internodes (Fig. 9). OCT offers the advantage of rapid in vivo measurements of internal stem properties across the entire stem length which may be important for overall lodging resistance in peas.

Whilst we believe the results are promising, this study was a proof-of-concept based on a limited number of plants for in vivo measurements. To accurately analyse the relationship between stem traits, such as stem wall thickness, stem width, and lodging resistance for different cultivars, future studies with larger sample sizes are required. As suggested previously, there is a trade-off between weight per unit length and stiffness [17] and this may impose a limit on stem wall thickness in natural populations of peas. Pea breeders are attempting to increase stem strength in peas beyond what exists in natural populations [15], and therefore our results are important because they permit selection for improved stem strength nondestructively in pea stems during the growing season.

In addition, mechanical properties of stem tissue are critical factors that could contribute to stem strength, as such, traits related to the geometry of the stem structure are not the only considerations. To analyse the mechanical properties of stem tissue, functional OCT techniques such as optical coherence elastography [35–37] and OCT-based optical palpation [38] could be utilised to measure the strain, stress and stiffness of the stem tissue in living plants. Combining all these measurements may provide a comprehensive analysis of the factors that determine stem strength and lodging resistance in living plants.

In this study, we have demonstrated the feasibility of in vivo imaging of living plants in pots using a benchtop OCT system. However, we acknowledge that transportation of the plants from the field station to the laboratory may introduce environmental changes that could potentially affect stem traits and add measurement errors in lodging resistance analysis. To mitigate this issue, a more straightforward and rigorous approach would be to measure traits, such as stem wall thickness and stem width, in plants growing in the field or glasshouse. This would require the development of handheld OCT probes and portable OCT systems. Handheld OCT probes have been previously demonstrated in other application areas, incorporating miniature optical components such as microelectromechanical systems (MEMS) and gradient-index (GRIN) lenses [39–41]. To facilitate the portability of OCT systems, one key enhancement is to develop a fully batterypowered system [42]. This advancement would eliminate the dependence on external power sources and significantly improve the manoeuvrability and flexibility of the OCT system during data acquisition in various field settings. With the use of compact handheld probes and portable OCT systems, in vivo measurement of stem traits can be performed at different stages of plant growth, enabling monitoring of growth patterns and lodging-resistance phenotypes throughout the life cycle of the plants. This information is critical for breeding and selecting the optimal cultivars that exhibit resistance to lodging and have high yield potential.

## Conclusions

Our study has demonstrated the efficacy of OCT for accurately measuring the stem wall thickness of live field pea. In comparison to ex vivo measurements, the* in vivo* measurements resulted in an average error of only − 3.1% across 20 imaged plants. We further investigated stem wall thickness along different internode positions in two field pea cultivars, Dunwa and Kaspa. The results showed that Dunwa had a uniform stem wall thickness across different internode positions, while Kaspa had a significantly negative slope of − 0.0198 mm/node. Both cultivars exhibited an increase in stem width along the internode positions; however, Dunwa had a rate of increase three times higher than that of Kaspa. Based on our results, it can be concluded that there are significant differences in the stem structure and subsequently, the stem strength, between these two field pea cultivars. This study underscores the potential of OCT as a rapid and accurate tool for in vivo and non-destructive measurement of stem wall thickness. This information can aid in the characterisation of plant traits and in breeding of cultivars that are resistant to lodging. Moreover, this method can be extended to other crops with similar stem structures, facilitating the development of a range of new and improved cultivars.

### Electronic supplementary material

Below is the link to the electronic supplementary material.

Supplementary Material 1

Supplementary Material 2

## Data Availability

The datasets supporting the conclusions of this article are included within the article (and its supplementary files).
