# Supplementary material for: In vivo characterisation of field pea stem wall thickness using optical coherence tomography
Source: Plant Methods. 2023 Oct 11;19:105. doi: 10.1186/s13007-023-01075-1 (PMC10566190; doi:10.1186/s13007-023-01075-1)
Supplement: Supplementary file 1 — Supplementary Material 1 [file 13007_2023_1075_MOESM1_ESM.docx]

Supplementary

*S1. Effect of light beam refraction on the stem wall thickness measurement*

In this Supplementary section we analyse the effect of light beam refraction on the measurement of the stem wall thickness. Refractions of the light beams occur at the air‑stem interface when the stem under inspection is placed oblique to the incident beam, as shown in Fig. S1(a). In this figure, the angle of incidence is $\alpha$ and the angle of refraction is $\beta$. According to Snell’s law, the relationship between $\alpha$ and $\beta$ can be expressed as:

$$n_{air}\cdot\sin\alpha=n_{stem}\cdot\sin\beta, (1)$$

where the refractive index of the air $n_{air}=1$ and $n_{stem}$ is the refractive index of the stem tissue. We denote the physical thickness of the stem wall as $T_{0}$, and the physical path length $L$ that the refracted light beam travels between the outer and inner stem walls is:

$$L=\frac{T_{0}}{\cos\beta}. (2)$$

When imaging the stem wall with OCT, the sensor records optical signals at various lateral location. These signals represent the light scattered by the stem tissue along the path of the refracted light, as indicated by the red dashed line in Fig. S1(b). Without the correction for refractive index of the stem tissue (assuming $n=1$ for the stem tissue), the optical path length of the refracted light propagating through the stem wall is:

$$L_{opt}=L\cdot n_{stem}. (3)$$

During the stem wall thickness measurement using the ThorImage software, a correction for the refractive index of the stem tissue was applied to ensure accurate results. This correction involves scaling the distance along the direction of the incident beams by a factor of $n_{stem}$, as depicted in Fig. S1(c). In this figure, the refracted light propagating through the stem wall has a measured path length of $L$, representing the physical path length of the stem tissue along the direction of the refracted beam, as described in Eq. (2).

Due to the scaling in the axial direction, the angle of incidence appearing in the generated OCT image is denoted as $\gamma$, as shown in Fig. S1(c). The relationship between $\alpha$ and $\gamma$ is expressed as:

$$tan\gamma=\frac{tan\alpha}{n_{stem}}, (4)$$

and the stem wall thickness to be measured using the ThorImage software can be expressed as:

$$T=\frac{L}{\cos\gamma}. (5)$$

By substituting Eqs. (1), (2) and (4) into Eq. (5), the measured stem wall thickness $T$ can be expressed as:

Figure S1. Schematic diagrams of (a) Refraction of the beam passing through the air‑stem interface, (b) Generation of the stem wall OCT image without the correction for refractive index, and (c) Generation of the stem wall OCT image with the correction for refractive index.


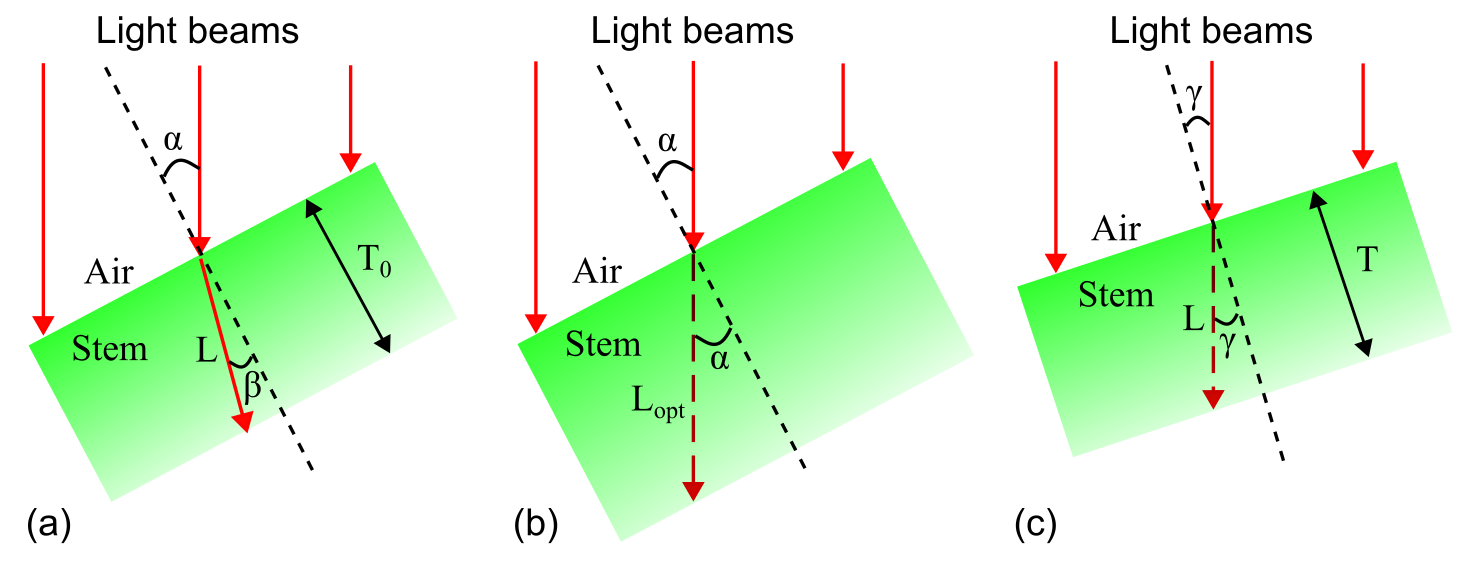

$$T=T_{0}\cdot\frac{\cos\left( \tan^{-1} \left( \frac{\tan\alpha}{n_{stem}} \right) \right)}{\cos\left( \sin^{-1} \left( \frac{\sin\alpha}{n_{stem}} \right) \right)}. (6)$$

According to Eq. (6), the measured stem wall thickness $T$ will slightly deviate from the physical thickness $T_{0}$ by a factor that is a function of the angle of incidence $\alpha$ (assuming $n_{stem}=1.33,$ as measured in the ‘Refractive index measurement’). For instance, if $\alpha=15^{\circ}$, $T=0.9994T_{0}$, $error=0.06\%$; if $\alpha=30^{\circ}$, $T=0.9899T_{0}$, $error=1.01\%$; if $\alpha=45^{\circ}$, $T=0.9437T_{0}$, $error=5.63\%$. However, for most of our *in vivo* OCT measurements, $\alpha\leq30^{\circ}$, making the effect of light beam refraction on the stem wall thickness measurement negligible.

In summary, the ThorImage software effectively corrects for the refractive index of the stem tissue during the measurement process, allowing for highly accurate stem wall thickness measurements. The minor deviations that may occur are mainly relevant when dealing with larger angles of incidence, which are typically not encountered in most *in vivo* OCT measurements.

*S2. Tables of raw measurement data*

All the raw measurement datasets are provided as tables in the file ‘Tables of Raw Data.xlsx’.

Table S1: Dunwa raw measurement data of stem wall thickness for *in vivo* validation.

Table S2: Kaspa raw measurement data of stem wall thickness for *in vivo* validation.

Table S3: Dunwa in vivo measurements of stem wall thickness and stem width at different internode positions.

Table S4: Kaspa in vivo measurements of stem wall thickness and stem width at different internode positions.

Table S5: Raw data of refractive index measurements.
